# Synthetic Cannabinoid use in a Case Series of Patients with Psychosis Presenting to Acute Psychiatric Settings: Clinical Presentation and Management Issues

**DOI:** 10.3390/brainsci8070133

**Published:** 2018-07-14

**Authors:** Stefania Bonaccorso, Antonio Metastasio, Angelo Ricciardi, Neil Stewart, Leila Jamal, Naasir-Ud-Dinn Rujully, Christos Theleritis, Stefano Ferracuti, Giuseppe Ducci, Fabrizio Schifano

**Affiliations:** 1 Highgate Mental Health Centre, Camden & Islington NHS Foundation Trust, London NW1 0PE, UK; antonio.metastasio@candi.nhs.uk (A.M.); angelo.ricciardi@candi.nhs.uk (A.R.); Neil1.Stewart@candi.nhs.uk (N.S.); leila.jamal@candi.nhs.uk (L.J.); naasir-ud-dinn.rujully@candi.nhs.uk (N.-U.-D.R.); 2 Department of Psychological Medicine, King’s College London, London WC2R 2LS, UK; 3 Department of Mental Health, ASL Rome 1, 00135 Rome, Italy; angelo.ricciardi@aslroma1.it (A.R.); giuseppe.ducci@aslroma1.it (G.D.); 4 Department of Psychosis Studies, King’s College London, London WC2R 2LS, UK; christos.theleritis@kcl.ac.uk; 5 National and Kapodistrian University of Athens, Eginition Hospital, 11528 Athens, Greece; 6 Department of Human Neurosciences, University of Rome ‘La Sapienza’, 00185 Rome, Italy; stefano.ferracuti@uniroma1.it; 7 Department of Psychopharmacology, University of Hertfordshire, Hertfordshire AL10 9AB, UK; f.schifano@herts.ac.uk

**Keywords:** synthetic cannabinoids, SCRAs, NPS, novel psychoactive substances, NPS testing, antipsychotics, mental health, physical health, nursing care, psychosis

## Abstract

Background: Novel Psychoactive Substances (NPS) are a heterogeneous class of synthetic molecules including synthetic cannabinoid receptor agonists (SCRAs). Psychosis is associated with SCRAs use. There is limited knowledge regarding the structured assessment and psychometric evaluation of clinical presentations, analytical toxicology and clinical management plans of patients presenting with psychosis and SCRAs misuse. Methods: We gathered information regarding the clinical presentations, toxicology and care plans of patients with psychosis and SCRAs misuse admitted to inpatients services. Clinical presentations were assessed using the PANSS scale. Vital signs data were collected using the National Early Warning Signs tool. Analytic chemistry data were collected using urine drug screening tests for traditional psychoactive substances and NPS. Results: We described the clinical presentation and management plan of four patients with psychosis and misuse of SCRAs. Conclusion: The formulation of an informed clinical management plan requires a structured assessment, identification of the index NPS, pharmacological interventions, increases in nursing observations, changes to leave status and monitoring of the vital signs. The objective from using these interventions is to maintain stable physical health whilst rapidly improving the altered mental state.

## 1. Introduction

Recent statistics in England have reported an increased number of hospital admissions with a primary diagnosis of drug-related mental health/behavioural disorders and poisoning by illicit drugs, respectively of 12 and 40% compared to statistics released in 2006/07 [1]. Over the last five years deaths involving Novel Psychoactive Substances (NPS) have sadly increased, with a further 8% increase in 2016 [2]. NPS represent an emerging and concerning global phenomenon [3,4,5] mainly due to: (1) the difficulties posed in the clinical management, of both mental and physical health; and (2) the absence of a clear and formal/structured description of the clinical presentation of patients using NPS, with obvious repercussions on clinical management.

NPS are a heterogeneous class of typically synthetic molecules including: synthetic cannabinoid receptor agonists (SCRAs), synthetic cathinones, amphetamine-derivatives, psychedelic phenethylamines, ketamine derivatives, novel tryptamines, synthetic opioids and sedatives (GABA-A/B agonists) [3,6,7]. Trends in illicit drug use over the last decade clearly show that adolescents and young adults give preference to NPS instead of traditional psychoactive substances (TPS) (e.g., cannabis, cocaine, heroin, amphetamines, LSD, ecstasy, ketamine, etc), because NPS are cheap and easily available either on the street or from websites [6,7,8]. They are difficult to identify in blood or urine drug screening (UDS) [9]. Lifetime NPS consumption was reported by 8% of young individuals in 2015 [10] up from 5% in 2011 [6]. Young adults (aged 16 to 24) are around twice as likely to have used an NPS in their lifetime compared to older adults (aged 16 to 59) [11]. It has recently been reported that the three psychiatric diagnoses most frequently associated with NPS use are bipolar disorder (23.1%), personality disorders (11.8%), and schizophrenia and related disorders (11.6%) [12].

In comparison to TPS users, poly NPS users are more likely to be young males [11], with daily use of traditional cannabis, weekly or more use of ecstasy, recent LSD use, higher levels of poly drug use, and a history of overdose on any drug in the past year [13]. Young adults attending nightlife events in pubs and discos are also more prone to poly-substance use, mainly combining NPS with alcohol and cocaine [14]. NPS users also tend to have a forensic history and a history of promiscuous sexual activity (e.g., chem-sex) [13].

Few studies have attempted to provide a psychopathological description of the clinical presentation of NPS users in acute settings. For example, in an observational cohort study enrolling consecutive adult patients presenting to an Emergency Department (ED) in London, the most common clinical features identified were seizures and agitation [15]. In a recent study looking into the impact of NPS misuse on admissions to an acute psychiatric facility in London, increased levels of violence in the group of NPS users were identified ([16]. Data collected in the Accident and Emergency departments (A&Es) of ten European countries have shown that the association between NPS use and the occurrence of psychosis varied considerably, depending on the type of drug used [17]. In particular, psychotic symptoms were noted in 6.3% of a large sample (5529 consecutive cases), with psychosis being more common amongst NPS users that had used tryptamines, methylenedioxypyrovalerone (MDPV), methylphenidate, SCRAs and amphetamine-type compounds [17]. A mounting range of evidence suggests that SCRAs can trigger the onset of acute psychosis in vulnerable individuals and/or exacerbate psychotic episodes in those patients with a previous psychiatric history. The literature reports a wide range of psychopathological issues such as paranoid thoughts, increases in aggressive and combative behavior, together with confusion, agitation and suicidal thoughts [18]. It has been suggested that SCRAs may have a higher psychosis-inducing potential compared to natural cannabis [19] because of the lack of cannabidiol—a substance associated with the medicinal effects of cannabis. For a review on existing studies and models of cannabis induced psychosis see a review from Murray et al [20].

Professionals usually report feeling less confident about managing NPS compared to TPS users, specifically because of the lack of clear guidance regarding the clinical management and the increased risk of toxicity [10,11,12,13,14,15,16,17,18,21]. For example, with the ingestion of NPS with high serotonergic activity (e.g.: psychedelic phenethylamines), misusers may present with hyperthermia, seizures, and hyponatraemia. Conversely, NPS with high dopaminergic activity (e.g., methylphenidate-like drugs such as some synthetic cathinones) are highly addictive and associated with prolonged stimulation, insomnia, agitation, and psychosis [22,23]. Furthermore, most SCRAs are at times associated with medical emergencies such as hypertension, myocardial infarction, renal failure [24], elevated heart rate, hyperglycaemia, nausea, vomiting, hypokalaemia, and seizures [18]. Moreover, in view of the increased use of latest generation of sedatives or ultra-high potency fentanyl derivatives, the assessment of vital parameters is of paramount importance when NPS users are presenting to an emergency or acute facility [3,4,5,6].

The implementation of an appropriate and safe clinical management plan is commonly based on patients’ accounts on which kind of NPS have been used. Acute mental health and emergency services are not routinely equipped with urine drug screening tests (UDS) for NPS in order to identify and provide an appropriate toxicological confirmation [15]. This causes considerable limitations when offering a targeted treatment strategy that can address patients’ presentations and potentially life-threatening intoxications [9,13,25].

At present, there is a dearth of detailed information relating to the clinical presentation of NPS users in acute mental health settings, especially in terms of: (1) descriptions of behavioural and psychopathological features using structured assessment and psychometric scales; (2) analytical toxicology and identification of the index NPS used; and (3) appropriate and evidence-based clinical management plans. This results in a range of difficulties in formulating targeted/individually tailored treatment plans.

The aims of this case series are: (1) to offer a standardized description of NPS users’ clinical presentations to provide clinicians with objective and measurable clinical pictures aimed at shaping protocols and standard operating procedures when NPS use is suspected and/or detected; (2) to provide best practice advice in the management of mental state alterations and medical complications, with the goal of reducing the number of serious adverse outcomes associated with NPS use.

## 2. Materials and Methods

### 2.1. Study Design and Recruitment

Data on presentation and clinical management of 4 cases, selected amongst patients consecutively admitted to two acute psychiatric wards from June 2017 to June 2018 at Highgate Mental Health Centre—Camden & Islington NHS Foundation Trust—were retrospectively collected using a database. Patients were aged 18 and 65 years and admitted because of presenting with psychotic illnesses and with a history of NPS use before or during admission. Data were collected using a standardized database to capture a range of information at baseline i.e., the time of the admission, and then during and after NPS intake. The information collected revolved around: (1) the clinical presentation, with a formal description of the psychotic symptoms; (2) the type of recreational drug(s) used; (3) the physical health outcomes; (4) the levels of psychiatric inpatient observation and leave status.

The clinical presentation was identified with the help of clinical notes and corroborated by the retrospective scoring of the Positive and Negative Syndrome Scale (PANSS), a multi-item questionnaire widely used to quantify disease severity in schizophrenia and psychosis [26]. Monitoring data of vital signs were collected using the National Early Warning Signs tool (NEWS) [27]. Data on the type of substance used were collected using routine drug screening tests for traditional psychoactive substances whilst a more thorough analysis (urine and/or oral fluids tests; Alere Laboratories technologies) was carried out to identify the index drug(s) used by both NPS and TPS patients. All subjects identified received medications included in the British National Formulary (BNF), such as benzodiazepines (BZO) and antipsychotics (first and second generation), with the choice guided by the clinical presentation and the drug testing results. Levels of nursing care, such as close or general observations, were also recorded together with the possibility of leave in the hospital grounds, either accompanied by staff (escorted) or alone (unescorted).

### 2.2. Participants

#### 2.2.1. Inclusion Criteria

Cases were selected amongst patients with recent or current histories of NPS use, aged 18–65 years, presenting to acute services with a psychotic illness classified by the International Classification of Diseases, Mental and Behavioral Disorders [28] as schizophrenia, schizotypal, delusional, and other non-mood psychotic disorders (ICD-10 codes: F20-29), mood (affective) disorders (ICD-10 codes: F30-39), and mental and behavioral disorders due to psychoactive substances (ICD-10 codes: F10-19). 

#### 2.2.2. Exclusion Criteria

Patients were excluded from the study if: (1) the psychotic symptoms were precipitated by an organic cause; (2) they had moderate or severe learning disability; (3) they suffered from a medical or neurological illness or (4) had an insufficient command of the English language.

### 2.3. Measures 

#### 2.3.1. Psychometric Measures

The Positive and Negative Symptoms Scale (PANSS) is a multi-item questionnaire widely used to quantify disease severity in schizophrenia and to assess the severity of positive (or productive) and negative (or deficit) symptoms of psychosis [26]. PANSS is easy to administer and is based on the clinician's interview with the patient; data are gathered looking at the patient's mental state over the previous week, with the patient's family and/or his/her acquaintances being able to provide further information. PANSS consists of 30 items and takes 45–50 min to be completed by the clinician.

The National Early Warning Score (NEWS; Royal College of Physicians, London, UK) [27] is a standardized system for the assessment of acute illness in adults. It is based on six vital signs such as respiratory rate, oxygen saturation, temperature, blood pressure, pulse/heart rate, and alertness. Each parameter yields a score of between 0 and 3 so that when the scores for all the parameters are summed a total NEWS score of between 0 and 18 is achieved. A score of 7 or greater indicates that a patient is likely to be critically medically unwell.

#### 2.3.2. Laboratory Measures

Standard urine drug screening tests were used to detect TPS such as heroin, cocaine, amphetamine, THC, and methadone. For NPS, the Alere drug screening urine and oral fluids tests were used. The urine tests provided a rapid screening of 30 synthetic cannabinoids at once whilst the oral screening test was able to detect mephedrone. 

## 3. Clinical Vignettes

### 3.1. Case 1—Mr A

28 years old Caucasian male, single and unemployed, living alone, with a positive forensic history and a diagnosis of Paranoid Schizophrenia. The patient had a 4 years’ history of psychosis with frequent relapses (5 admissions in 4 years). He was transferred to an acute treatment ward from a psychiatric intensive care unit (PICU). At the time of the transfer the patient was stable and on treatment with Risperdal Consta 37.5 mg fortnightly + Olanzapine 10 mg daily + Pregabalin 100 mg daily. The PANSS score was 73/210 and his psychopathology was mainly characterized by positive symptoms: delusional mood, persecutory and grandiose delusions and second and third person auditory hallucinations. The UDS was initially negative but, one week after the transfer, Mr A’s mental state deteriorated suddenly and he became very agitated and verbally and physically aggressive. He presented with a bizarre and repetitive behaviour consisting of stopping and remaining immobile for a few minutes and then running fast along the ward corridor. He also had second and third person auditory hallucinations, persecutory delusions and thought disorganization. He started to fear the hospital ward’s electronic fire alarms. He believed that the fire alarms were cameras that were spying on him and he was very preoccupied with specific members of the staff whom he believed were there to kill him. The hallucinations also became very severe and he was responding to internal stimuli constantly throughout the day. The total PANSS score was 109/210 and the UDS was positive for SCRAs. We decided to increase Olanzapine to 20 mg, daily and to add Clonazepam 8 mg, daily to manage the agitated behaviour and the psychotic symptoms. We also increased the level of monitoring of his vital measures by completing the NEWS scores twice a day. NEWS scored 2 with increased heart rate and fluctuating blood pressure. We considered a transfer to PICU but since the patient was starting to respond well to the new treatment plan and the reason for the relapse was evident (NPS intake), we decided to continue to treat the patient on the acute ward. Mr. A responded well to the change/increase of medications, his symptoms improved in 24 h and within 7 days from the acute intoxication the PANSS scores reduced to 74/210.

### 3.2. Case 2—Ms T

32 years old Caucasian woman, single and unemployed, living alone, with no forensic history and a diagnosis of Schizoaffective Disorder and poly-substance misuse (mainly crack cocaine and heroin). Ms T was stabilized on a combination of Aripiprazole 30 mg, daily + Lithium carbonate 800 mg, at night. The PANSS score was 95/210 and the UDS was negative for all illicit substances. Four weeks later, the patient’s mental state deteriorated suddenly. She became physically and verbally very aggressive with severe features of sexual disinhibition. The patient presented with delusional mood and with complex grandiose and persecutory delusions such that she believed she was part of a secret army and she had powers to kill people with her thoughts. She also believed she was being chased by the Albanian mafia and had to fight for her life. The patient also became very aggressive with members of staff and on four occasions it was necessary to call the emergency team to provide extra sedation. The PANSS score was consistent with the deterioration of her mental state, scoring 115/210 and the UDS tested positive for both SCRAs and THC. The clinical team felt that the patient needed a more robust pharmacological treatment plan and therefore Haloperidol 10 mg daily + Clonazepam 8 mg daily were added. The patient remained acutely unwell for more than 72 h. Ten days after the intoxication Ms T remained still irritable and agitated. The PANSS score was 115/210, 10 points higher than the baseline, and the UDS continued to test positive for SCRAs. NEWS were increased to twice a day but the score was always within range (0 or 1) with tachycardia being the only altered parameter. Meanwhile, other patients on the ward tested positive for SCRAs and it was suspected that Ms T was bringing SCRAs to the ward. At that stage, leave was suspended and a stricter search policy was enforced on the ward. The patient’s mental state improved further and ten days later her urine tests were negative for SCRAs.

### 3.3. Case 3—Mr Y

20 years old Black-Caribbean male, single and unemployed, living with friends and with no forensic history, was quickly re-admitted to a treatment ward following the sudden onset of bizarre behaviour after an earlier discharge from another ward. The diagnosis was First Psychotic Episode in the context of poly-substance misuse. On admission, Mr Y was on Haloperidol Decanoate 50 mg, monthly + Haloperidol 10 mg, at night (on reducing regime). He appeared severely thought-disordered, sexually disinhibited and aroused, approaching other patients for sex or suddenly becoming physically aggressive by spitting on others. The PANSS score was 116/210 with prominent positive symptoms (positive symptoms subscale 40/49). He presented as being severely disruptive, chaotic, and intrusive into other patients’ care, attacking staff and other patients, urinating on the floor and spitting at other people’s faces. Mr Y was therefore treated with Aripiprazole 9.75 mg three times a day + Clonazepam 6 mg daily in divided doses. Observation levels were increased to 2:1 arms’ length to reduce risks of retaliation from others due to sexually inappropriate and aggressive behaviour. NEWS monitoring was increased to hourly to monitor any possible deterioration in physical health. UDS were positive for benzodiazepines and SCRAs. The patient remained unwell. Observation levels were maintained at 2:1 arms’ length and NEWS monitoring decreased to TDS once physical outcomes remained stable for 12 h. After 72 h the clinical condition improved with a reduction of PANSS score to 98/210. Eventually, because of the continued high risk of retaliation from others Mr. Y was transferred to a Psychiatric Intensive Care Unit (PICU).

### 3.4. Case 4—Mr G

39 year old Asian British man, married and unemployed, living with his family and with a long forensic history. Mr G had a long-standing history of Bipolar Disorder since the age of 28. He had a history of numerous admissions, was non-compliant with his medications, and engaged poorly with his community team. He presented with a long-term history of poly-substance misuse (e.g., alcohol, cocaine, MDMA, cannabis, “legal highs”). He had previously been treated with a mood stabilizer (Sodium Valproate); Zuclopenthixol and Risperidone Depot (both stopped due to sexual dysfunction); Olanzapine and Quetiapine (both stopped due to poor response). At the time of his admission to Highgate Mental Health Centre, he was administered Abilify Depot 400 mg, monthly with no or little efficacy. He was transferred from another ward on Section 3 due to a manic relapse, with no leave and a diagnosis of Bipolar Affective Disorder (BPAD, current episode manic). Mr. G had a long history of violence towards staff and patients (he broke a nurse's nose and stabbed another patient with a pen). At the time of the admission, he was very agitated, aggressive and intimidating, banging his fist on the table and threatening staff with a glass bottle. He also showed bizarre behavior, e.g., wearing sunglasses whilst indoors, holding pieces of paper with some incomprehensible notes on Hitler, quantum physics and aliens. He was thought disordered with grandiose delusional beliefs regarding him being the King of Egypt and able to cause a nuclear war. It proved very difficult to verbally de-escalate him and he did not agree to change his medication regime as he believed that he should be treated “only with love”. The PANSS score was 108/210. Abilify was withdrawn and Zuclopenthixol started whilst he continued the rest of his medications. On admission, UDS was negative for both NPS and TPS. A week later, UDS was positive for both benzodiazepines and SCRAs, and NEWS was increased to TDS. Two weeks later, UDS continued to be positive to SCRAs, no changes to his leave status were made, with garden leave being maintained. Three weeks after admission, UDS was positive for benzodiazepines and THC and four weeks later the admission UDS was positive for THC and SCRAs. After admission, his mental state remained unsettled with refractory manic positive symptoms and a poor response to medication. The PANSS score was 123/210. Hence, his leave was stopped and, a week later, his UDS became negative for all substances, SCRAs included. His positive symptoms started to improve with a reduction of PANSS to 66/210. Over the following four weeks Mr. G appeared well kempt and settled on the ward, with no grandiose delusions and no further episodes of aggression. He showed a satisfactory response to Zuclopenthixol 300 mg, weekly + Sodium Valproate 1200 mg. UDS was negative for all substances and, therefore, Mr. G was safely discharged to the community team.

## 4. Discussion

NPS misuse is a recent phenomenon and knowledge of its effects, either in the short or the long term, on the population is relatively poor [3]. There is an increasing amount of knowledge regarding the effect that NPS have on individuals with severe mental illness [12]. However, well documented evidence of the negative impact are limited and most cases have not been corroborated by analytic chemistry evidence of the NPS used [15]. Furthermore, there is a paucity of data to guide the monitoring and management of patients with severe mental illness who take NPS, and then suffer from acute psychopathology and physical ill-health [3].

To the best of our knowledge, our case series is the first and only attempt made at describing, with the use of a specific psychometric scale (PANSS), the effects of acute intoxication with NPS (mainly SCRAs) identified through UDS in an acute hospital setting in patients suffering from severe psychotic disorders. All four cases show how clinically significant the impact of SCRAs use was on their mental states. The cases showed marked and sudden clinical deteriorations, with intense exacerbations of positive symptoms, psychomotor agitation, sexual disinhibition, verbal/physical aggression, and poor responses to medications. The latter phenomenon makes the clinical and risk management of these patients more difficult, and it is therefore necessary to develop appropriate management plans to minimize such risks.

In order to set up an appropriate and safe Informed Clinical Management Plan (ICMP) (Table 1), the first step advised here is to establish, whenever possible, which NPS is responsible for the intoxication due to their wide range of effects. We advocate, therefore, the use and further development of reliable drug tests to identify the specific NPS types associated with particular clinical presentations. Accurate testing for NPS would assist in establishing clear diagnoses, formulating ICMPs and identifying the most effective treatments for intoxications with particular NPS. 

The cases described here were all characterized by acute SCRA intoxications. The clinical presentations were characterized by an acute onset of agitation and aggressive behavior; the symptoms decreased in intensity and frequency in no less than 72 h. The management of acute intoxications was by identifying the substances responsible for the sudden deteriorations, and by treating the symptoms with benzodiazepines and antipsychotic medications. In addition vital parameters were monitored, nursing observations were increased, and leave statuses were changed. These measures led to rapid and successful resolutions of symptoms and reduced the need for transfer to more intensive care settings. Furthermore, they promoted more rapid step-down and recovery in the community.

In general terms, pharmacological treatment remains the mainstay of treatment. However, the novelty of the use of medications according to our protocol is that pharmacological interventions are guided by NEWS and toxicology results. For example, haloperidol should be avoided in patients that have used cathinones for the toxic effect on cardiac rhythm; and benzodiazepines should be avoided in patient with a NEWS score of 3 because of low oxygen saturations levels. 

In terms of pharmacological treatment, the use of BDZ has been recommended with or without a second-generation antipsychotic (SGA) to reduce the risk of cardiac side effects [22,23]. Benzodiazepines remain the first line treatment, although their use needs to be weighed against the risk of respiratory depression when given to subjects who have ingested alcohol and/or unknown substances [6,7,8,9,10,11,12,13,14,15,16,17,18]. Amongst the SGAs, whilst aripiprazole is probably the safest antipsychotic to be used in such scenarios because of its negligible effect on QTc, olanzapine has proven to be effective in treating psychotic symptoms caused by NPS [29].

## 5. Limitations

We are aware that four cases are not representative of the multi-faceted spectrum of presentations with NPS use/intoxication and observation of a wider sample size is necessary. However, we believe that this study is important in generating hypotheses that may lead to more comprehensive projects such as case control studies.

Furthermore, although the four cases presented were objectively described by using the PANSS to provide an objective measurement of the clinical observation, this was made retrospectively. We are also aware that the patients described were intoxicated with SCRAs. No other NPS such as cathinones or mephedrone were detected in our sample population. Therefore, our clinical description-albeit exhaustive and comprehensive-is limited to a subgroup of NPS users using only SCRAs. Moreover unfortunately, the UDS screening tests that were available were not able to identify with higher specificity the type of SCRAs used. 

It is worth noting how in terms of diagnostic categories our patients sample was not homogenous as one of the four patients was presenting with bipolar disorder. This may be an additional confounding factor; and, in a large enough sample, patients should be divided according to diagnoses. 

Finally, a randomized controlled trial (RCT) to establish which ICMP is necessary to establish which treatment plan is most effective in the management of individuals with a severe mental illness intoxicated with NPS would be helpful.

## Figures and Tables

**Table 1:** Description of the Camden & Islington—Informed Clinical Management Plan (ICMP).

Camden & Islington—Informed Clinical Management Plan (ICMP)	
MENTAL STATE assessment	
Using a psychometric scale (PANSS)	Monitoring mental state
NPS detection	
Using specific analytic toxicology to detect NPS (UDS and/or oral swabs)	Monitoring access to substances and leaves (reduced/suspended—escorted/unescorted)
MEDICATION & PHYSICAL HEALTH monitoring	
Using benzodiazepines (BDZ) and/or second generation antipsychotics (SGA) when possible	Monitoring physical health (NEWS) Levels of nursing observations
